# Chirality-Specific Unidirectional Rotation of Molecular Motors on Cu(111)

**DOI:** 10.1021/acsnano.2c12720

**Published:** 2023-02-09

**Authors:** Monika Schied, Deborah Prezzi, Dongdong Liu, Stefan Kowarik, Peter A. Jacobson, Stefano Corni, James M. Tour, Leonhard Grill

**Affiliations:** † Department of Physical Chemistry, Institute of Chemistry, University of Graz, Heinrichstraße 28, 8010 Graz, Austria; ‡ Nanoscience Institute of the National Research Council (CNR-NANO), via G. Campi 213/a, 41125 Modena, Italy; § Departments of Chemistry and Materials Science and NanoEngineering, the Smalley Institute for Nanoscale Science and Technology, the Welch Institute for Advanced Materials and the NanoCarbon Laboratory, Rice University, Houston, Texas 77005, United States; ∥ Dipartimento di Scienze Chimiche, Università di Padova, Padova I-35131, Italy

**Keywords:** molecular motors, unidirectional rotation, chirality, scanning tunnelling microscopy, nano machines, single-crystal surface, adsorption

## Abstract

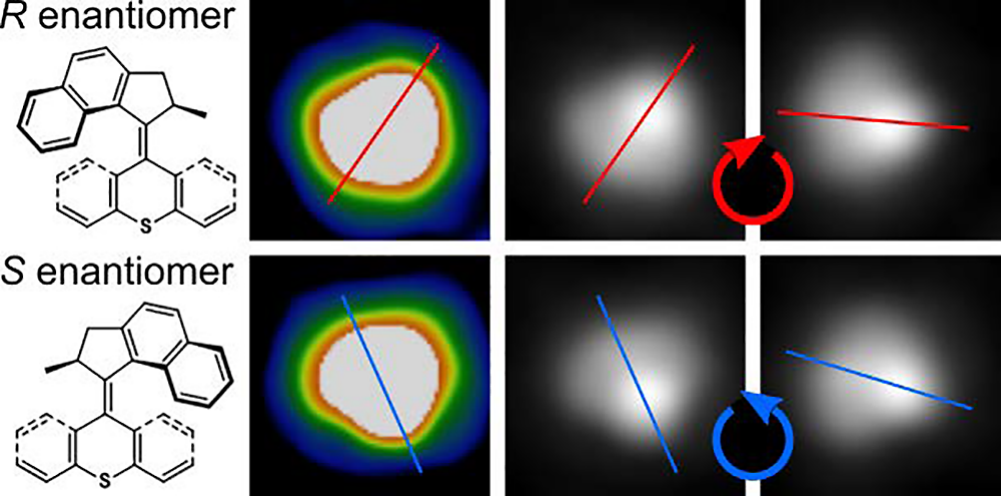

Molecular motors have chemical properties that enable unidirectional motion, thus breaking microscopic reversibility. They are well studied in solution, but much less is known regarding their behavior on solid surfaces. Here, single motor molecules adsorbed on a Cu(111) surface are excited by voltages pulses from an STM tip, which leads to their rotation around a fixed pivot point. Comparison with calculations shows that this axis results from a chemical bond of a sulfur atom in the chemical structure and a metal atom of the surface. While statistics show approximately equal rotations in both directions, clockwise and anticlockwise, a detailed study reveals that these motions are enantiomer-specific. Hence, the rotation direction of each individual molecule depends on its chirality, which can be determined from STM images. At first glance, these dynamics could be assigned to the activation of the motor molecule, but our results show that this is unlikely as the molecule remains in the same conformation after rotation. Additionally, a control molecule, although it lacks unidirectional rotation in solution, also shows unidirectional rotation for each enantiomer. Hence, it seems that the unidirectional rotation is not specifically related to the motor property of the molecule. The calculated energy barriers for motion show that the propeller-like motor activity requires higher energy than the simple rotation of the molecule as a rigid object, which is therefore preferred.

Molecular motors are fascinating objects that transform an external stimulus into useful motion.^1,2^ Dominated by thermal Brownian motion and viscous forces, wherein inertia becomes irrelevant, they are not simple analogues of their macroscopic counterparts.^3^ The small size of molecular machines is key as they are truly active at the atomic scale and are by nature compatible with other molecules, *e*.*g*., in biological matter,^4^ holding great promise for molecular nanotechnology.^1,5^ Artificial molecular motors have seen impressive developments,^1,6−9^ mainly caused by great achievements in synthetic chemistry^10−12^ and theoretical descriptions.^13^

For the control of molecular machines, it is important that the motor exhibits only one sense of rotation, resulting in unidirectional translation of the molecular machine, *i*.*e*. only forward and not backward motion, in contrast to random motion in all directions.^1^ Note that “motor” molecules differ conceptually from “rotor” molecules that can be rotated on a surface by the tip of a scanning tunneling microscope (STM).^14−18^ This is achieved either by a local deformation of the potential energy surface, for instance as a result of the electric field caused by the STM tip,^17^ or by an asymmetric sawtooth-shaped potential energy landscape and a nonthermal molecular excitation out of equilibrium.^18^ Such rotors can show a preferred sense of rotation,^15,17,18^ but they lack a (motor) unit that intrinsically transforms energy input into intramolecular motion of predefined direction.

An important class of molecular motors are the so-called Feringa motors, which consist of two sections: the stator and the rotor.^19^ The latter rotates in one way only with respect to the stator, exhibiting rotational frequencies in the MHz regime in solution.^20^ Feringa motors have been intensely studied in solution,^21−23^ but also in environments with a degree of confinement such as on gold nanoparticles,^24^ in metal–organic frameworks^25^ or at cell membranes.^26^ Few studies exist on such molecules on flat single-crystal surfaces, where two-dimensional confinement and well-defined adsorption configurations provide a frame of reference for molecular motion and dynamics.^27−31^ This approach is attractive as it allows precise characterization of the pathways on the surface, including local defects, and to follow individual molecular trajectories with high spatial resolution, making the observations statistically sound, by using STM at low temperatures. However, the surface must be considered, since substantial molecule–surface interactions as on metals can modify the potential energy landscape experienced by the molecule. This can completely change the relative stabilities of the various motor conformations.^31^

Chirality plays an important role in the function of molecular motors.^19,32^ Chemical synthesis results in both (*S*)- and (*R*)-enantiomers, which show different helicities, (*M*) and (*P*).^33^ An important step in the motor activity is the thermal relaxation of the helical structures, *i*.*e*. helix inversion, which occurs in one direction only. Thus, it is the presence of stereocenters in the molecules that are responsible for unidirectional movement. Note that the presence of a pseudoasymmetric center in achiral motor molecules can also cause unidirectionality.^34^ As a consequence of the motor’s chiral properties, different enantiomers of molecular motors rotate in opposite directions.^35^ In experiments, this property can be difficult to study as the synthesis of motor molecules generally leads to a racemic mixture, that is, an equal proportion of both enantiomers. Here, we study the dynamics of single molecular motors on a Cu(111) surface by low temperature STM. Consequently, we can correlate the dynamics of each molecule with its chirality.

## Results and Discussion

**Figure 1:**
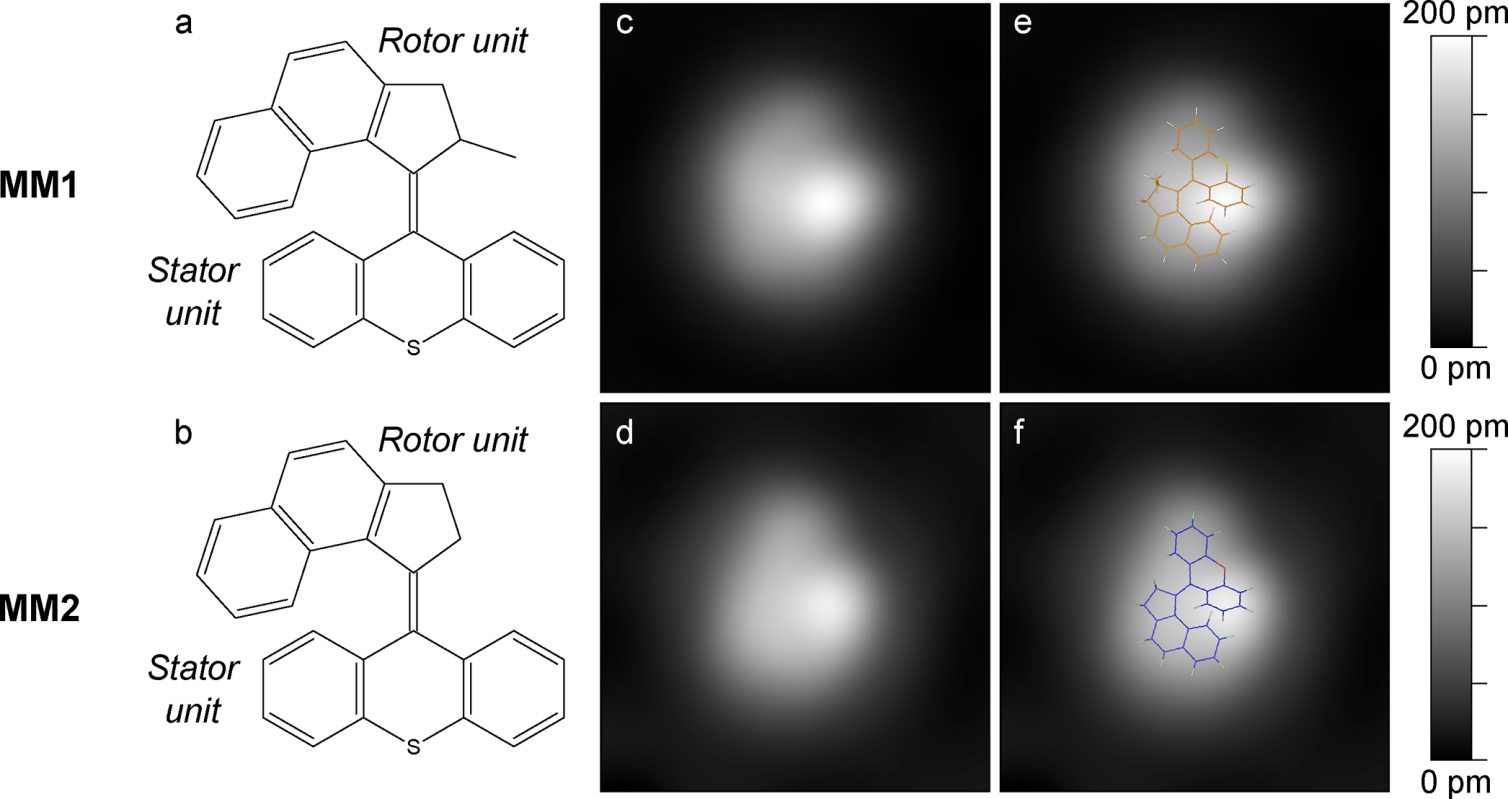
(a, b) Chemical structures of the MM1 (a) and MM2 (b) molecules (with the so-called rotor unit at the top and the stator unit at the bottom). (c, d) STM images (size: 3 × 3 nm^2^) of single MM1 (c) and MM2 (d) molecules on a clean Cu(111) terrace (tunneling parameters: (c) 183 mV and 186 pA, (d) 30 mV and 30 pA). (e, f) The same STM images as (c, d) with the molecular structure overlaid.

Two motor molecules were investigated on Cu(111), MM1^31^ and MM2 (abbreviation for molecular motor 1 and 2, respectively; Figure 1a,b), which exhibit different light-induced molecular dynamics in solution. MM1 provides unidirectional rotation; once in the excited state, the rotor becomes orthogonal to the stator, and the two directions of possible relaxation are diastereomeric, due to the adjacent stereogenic center at the methyl-attachment site and therefore differing in energy. The excited molecule proceeds over the lower energy barrier direction from that photochemical excitation. This is repeated after the thermal inversion step, thereby proceeding to a unidirectional movement. Conversely, MM2 has no methyl group and is devoid of an adjacent stereogenic center, resulting in no preferential direction of relaxation and a flapping-like action rather than unidirectional rotation. We therefore use MM2 as the “control molecule” in our study.

After deposition, the molecules MM1 and MM2 appear very similar (Figure 1c,d) as their chemical structures are closely related. The main difference in appearance is the slight protrusion next to the brightest lobe (at the left edge of the molecule in Figure 1c) for MM1, which is missing for MM2 (Figure 1d). With the molecular structures overlaid on the STM images (Figure 1e,f) it becomes evident that this protrusion is due to the methyl group (as determined by DFT calculations^31^) that is absent for MM2. As a consequence of the chiral chemical structure, the molecular appearance in the STM images is chiral and we find both enantiomers with the same abundance on the surface. As the MM1 methyl group stands upright,^31^ it has a limited effect on the molecule–surface interaction, which renders the adsorption geometries of the two molecules (MM1 and MM2) very similar, as confirmed by simulated images (see Figure S4). Note that this changes if deposition is done onto the slightly cooled sample (below about 290 K) where MM1 molecules also adsorb with the methyl group pointing downward, *i*.*e*. toward the surface.^31^ In agreement with the absence of the methyl group, MM2 does not exhibit this alternative conformation, not even for preparation at low sample temperatures. Hence, both molecules adsorb in one, very similar, conformation if deposited at room temperature, and this is our focus in the following.

**Figure 2:**
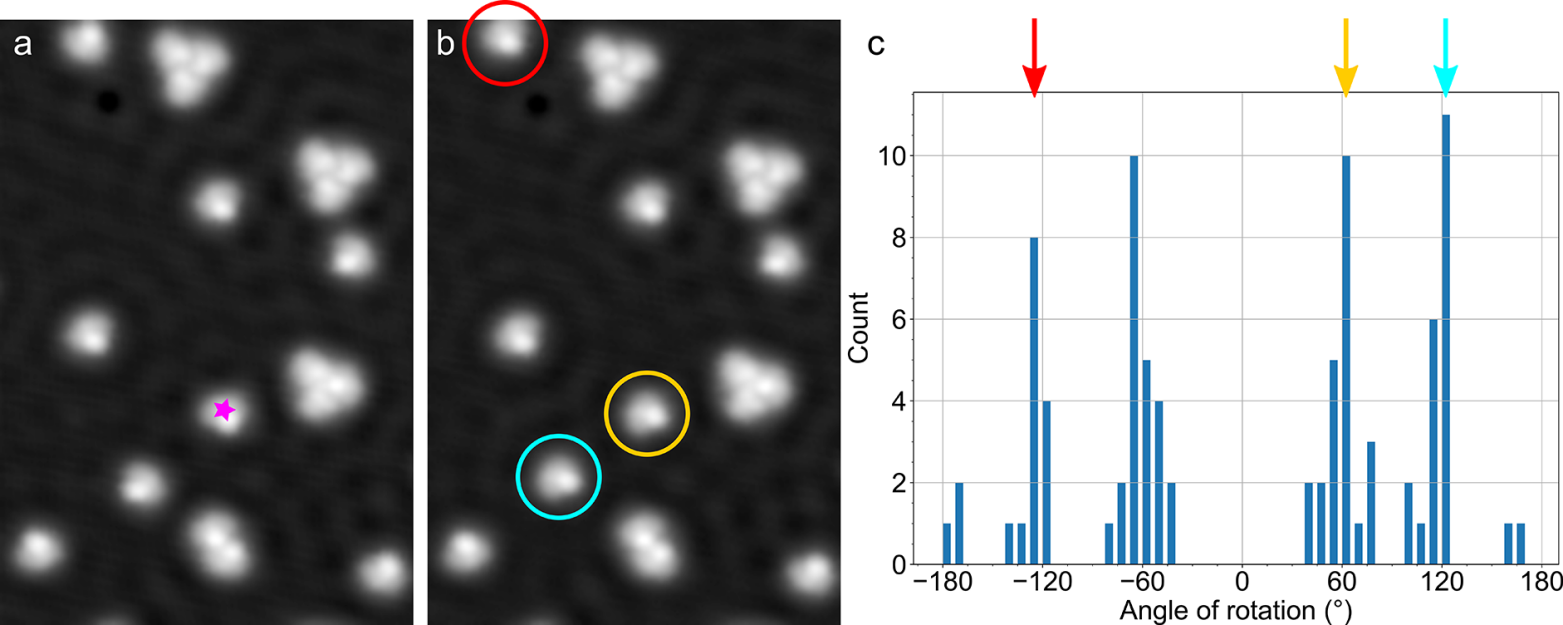
STM images (size: 14 × 20 nm^2^, −100 mV, 60 pA) of MM1 molecules (a) before and (b) after a voltage pulse from the STM tip (−0.85 V; position indicated with a star in (a)). The circles indicate three molecules that have rotated between the two images. (c) Histogram of molecular rotations for different rotational angles. The red, yellow, and cyan arrows indicate the rotational angles observed in (b) for the molecules circled with the corresponding color.

While STM can be used to image and manipulate single molecules, the proximity of the STM tip to the molecule of interest can affect the potential energy landscape of the molecule via short-range chemical forces or the local electric field.^36,37^ This can result in molecular motion on the surface, even without a motor unit. Consequently, to access the intrinsic dynamics of the motor it is necessary to either rigorously minimize interactions between the tip and the molecule or to activate dynamics in the motor remotely. Here, we opt for the latter and stimulate molecules that are laterally displaced from the STM tip (Figure 2). After imaging a region of interest (Figure 2a) with several isolated MM1 molecules (as well as some dimers), a voltage pulse (−0.85 V) is applied with the tip being in a fixed position (indicated by a star in Figure 2a). Following the pulse, the region of interest is imaged again, showing that several molecules (indicated by circles in Figure 2b) have rotated (translation is observed very rarely). Molecular rotation happens not only underneath the tip but also—and predominantly (>90% of the cases)—remotely (with the tip being laterally displaced from the molecule). The molecular shape remains the same before and after rotation, and the molecule retains its chirality; only the orientation with respect to the surface is changed. It should be noted that the molecular behavior is the same for both processes. Importantly, the remote rotation offers the possibility to analyze the process without deformation of the local potential energy surface, which can hardly be avoided for molecules underneath the STM tip.

**Figure 3:**
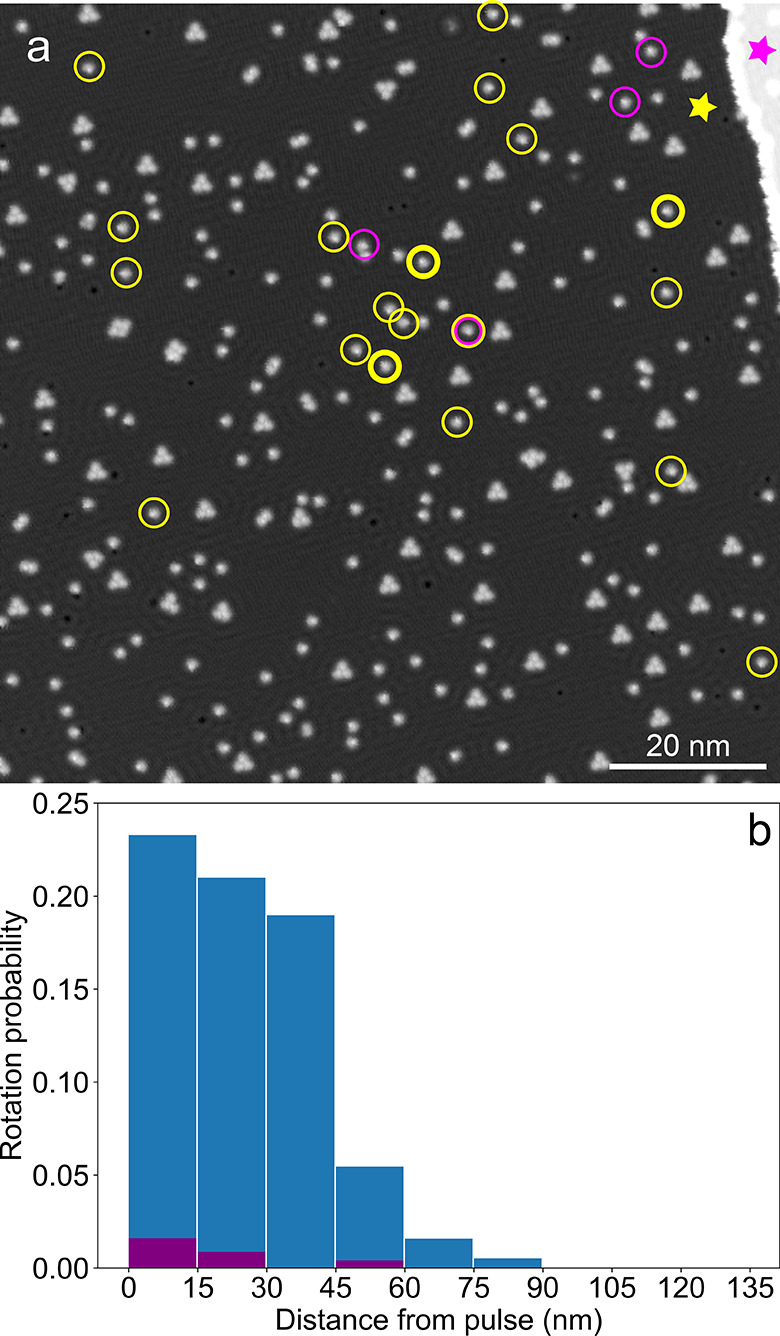
(a) STM image (size: 100 × 100 nm^2^, −100 mV, 60 pA) of MM1 molecules on Cu(111) with the STM tip positions of two pulse series, either on the lower (indicated by a yellow star) or the upper terrace (pink star), next to a step edge. All molecules that were found to rotate after 19 (21) pulses on the lower (upper) terrace are indicated by yellow (pink) circles, respectively. Molecules with bold circles rotated twice. (b) Histograms of the probability of a rotation event as a function of the lateral distance of the rotating molecule from the STM tip position during the pulse. The histograms are done for voltage pulses (indicated by stars in (a)) on the lower (blue columns) or upper (pink) terrace and are normalized on the number of molecules at each distance from the tip (see Supporting Information for details).

For STM-induced molecular manipulations, it is typical that the tip is placed directly above the molecule of choice, ensuring the molecule experiences both the maximum electric field strength and tunneling current (inducing inelastic scattering events). Here, molecules are also rotated if the STM tip is laterally shifted, even at very large distances of ∼100 nm from the STM tip (Figure 3). As the excess tunneling current during the pulse is spatially localized beneath the tip apex, there are two remaining options for the underlying mechanism. First, even with the tip laterally displaced from the affected molecules, the enhanced electric field during the voltage pulse can still be used to induce molecular processes,^38,39^ but it is strongly reduced as compared to the molecule underneath the tip.^40^ Second, hot charge carriers (electrons or holes) injected from the STM tip can travel along the surface until they trigger a process via inelastic scattering, similar to tautomerization in porphycene on Cu(111).^41^ To determine the operative mechanism in the present case, we use a monatomic step edge of the Cu(111) that efficiently scatters surface electrons.^42^ This effect is used to distinguish electric field-induced processes from hot charge carrier processes by comparing voltage pulses applied at either the upper or the lower terrace next to a step edge.^41^ In the case of an electric-field-driven process, very similar manipulation rates are expected since the field strength at the molecule position hardly differs. If instead hot charge carriers are responsible, they are scattered at the step edge so that a molecule that is located on the lower terrace can only be activated efficiently if the tip is placed on the lower (and not the upper) terrace.

The results of this experiment (Figure 3) show that voltage pulses on the upper or lower terrace next to a step edge (indicated by a pink or yellow star, respectively) lead to very different rotation probabilities, despite their small lateral separation. While the process is rather efficient for a pulse on the lower terrace (23 events during 19 pulses, see yellow circles in Figure 3a), rotations are rarely observed if the STM tip is placed on the upper terrace during the pulse (4 events during 21 pulses, pink circles). We thus interpret the remote activation of our molecules as the injection of electrons or holes into the surface state of Cu(111), similar to the work of Ladenthin et al.^41^ For pulses on the lower terrace (yellow in Figure 3) the electrons/holes are traveling across a Cu(111) terrace with only few obstacles such as scattering at molecules. On the other hand, electrons/holes that are injected on the upper terrace are efficiently scattered at the Cu(111) step edge, a well-known process on this surface,^42^ before they can reach and excite molecules on the lower terrace. Accordingly, the rate of rotation is strongly reduced as compared to the former case (Figure 3b). Molecular rotation can be induced at both bias voltage polarities with a threshold of about 0.5 V applied to the sample while the STM tip is grounded. The process is more efficient at negative voltages, which were therefore chosen for most experiments.

The most important property of molecular motors is the unidirectionality of their motion. In the case of the Feringa motors, this refers to the rotation of the rotor part with respect to the stator unit of the molecules.^19^ If the molecules are adsorbed on a flat surface, this propeller-like rotation can be expected to be transformed into motion with respect to the surface, either rotation or translation (or both). In general, for surface-adsorbed molecules (without a motor unit), rotation typically exhibits the lower activation barrier and is thus preferred over translation if both motions are possible.^17,43^ In the case of motor molecules, the unidirectionality of rotation in the pure molecule (in the gas phase) should be transferred to the surface-adsorbed molecule. Thus, it is a key question whether the MM1 molecules rotate in a unidirectional manner.

**Figure 4:**
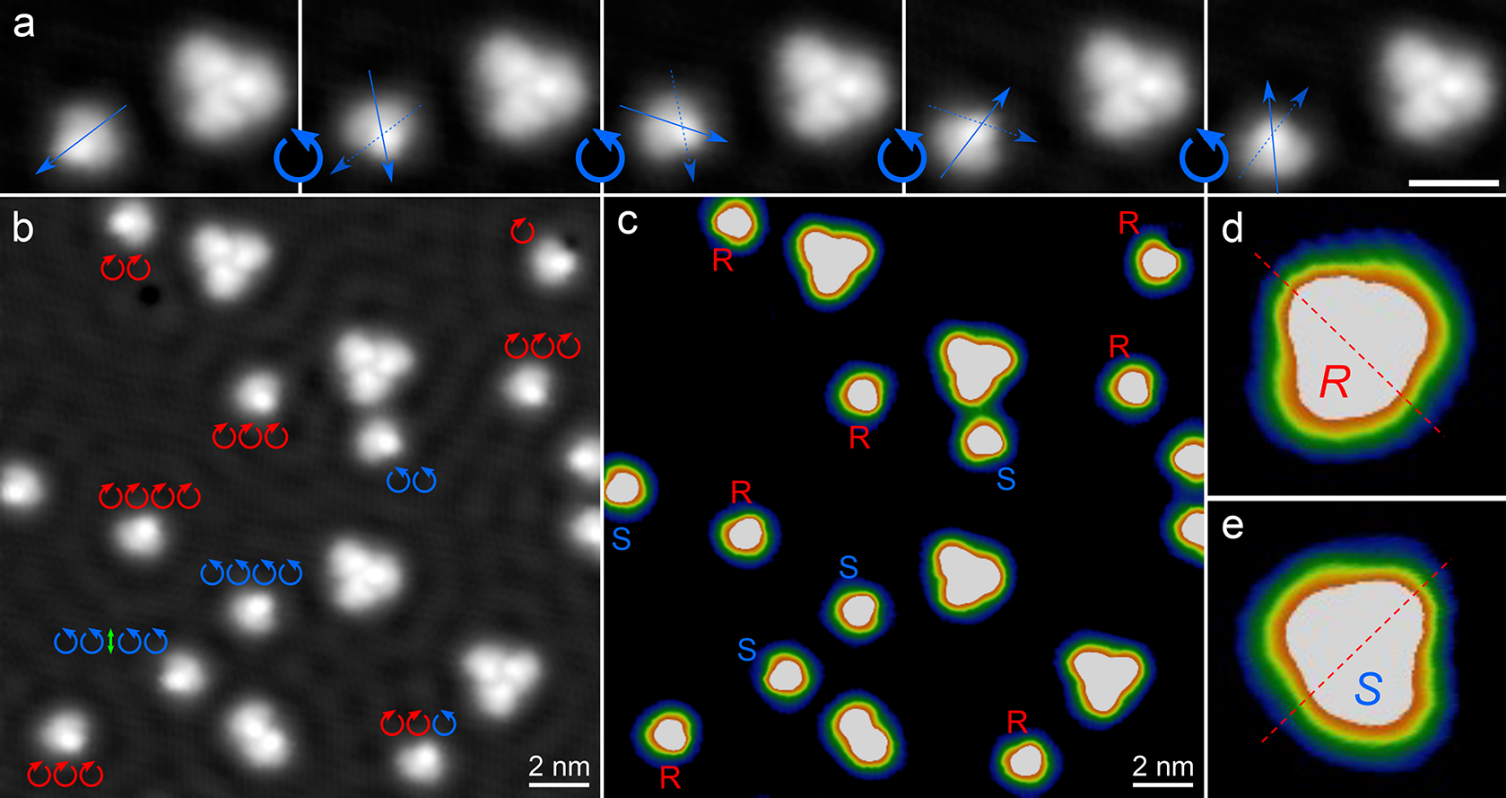
(a) Sequence of STM images (−100 mV, 60 pA, scale bar: 2 nm) of the same MM1 molecules on a terrace area on Cu(111). Dashed and solid blue arrows are superimposed on the molecule to indicate its previous and current orientation, respectively. The curved arrows between the images highlight the direction of rotation. (b–c) The same STM image (−100 mV, 60 pA) with different contrast scales to enhance the molecular chirality in (c). The directions of rotation are indicated in (b) for each molecule (the green double arrow at the bottom left indicates a rotation of 180° and thus an unknown direction) while the enantiomers are labeled in (c). (d and e) Zoom-in STM images (−50 mV, 60 pA, 2.26 nm × 2.26 nm) of single molecules that illustrate the asymmetric appearance that allows to identify individual *R*- and *S*-enantiomers, respectively.

In order to achieve a more precise measurement of the angle of rotation, many (*n* = 170) rotations from small-scale images such as Figure 4b from various surface areas on different sample preparations have been analyzed. Note that the tip shape does not affect the molecular behavior as tested by comparing molecular rotations before and after controlled tip indentation. This detailed analysis of many (*n* = 170) rotations (small rotations below 20° are neglected; see Supporting Information for details) reveals that the molecules have preferred rotational angles (Figure 2c), which are multiples of 60°. Consequently, peaks at around ±60°, ± 120°, and ±180° are observed, which reflects the sixfold symmetry of the Cu(111) surface. Note that angles of ±240° could in principle be incorrectly classified as ∓120°. However, as the number of events clearly decays with increasing angles and ±180° rotations are already very rare, it seems unlikely that this plays an important role. We find that 48% of the rotations are anticlockwise and 52% are clockwise. This near even distribution could suggest that there is no unidirectional motion. However, this is the average over many molecules and the situation changes substantially if individual molecules are analyzed one-by-one. When following a single molecule during a sequence of rotations (Figure 4a), we find that it continues to rotate in one direction only—in contrast to the averaging picture over many molecules. For instance, the molecule depicted in Figure 4a always rotates anticlockwise in four subsequent rotations (with +60° in each step). While this molecule rotates only anticlockwise, other molecules are found to do the opposite, namely repeatedly rotating clockwise. Therefore, the direction of rotation is characteristic for each individual molecule.

Figure 4b shows an overview image with various molecules that were rotated several times in a manipulation sequence. In agreement with the uniform statistics discussed above, both rotation directions occur in this group of molecules and the sense of rotation is indicated for each molecule in Figure 4b (clockwise: red, or anticlockwise: blue, 180°/unknown: green). Only when analyzing single molecules, not the ensemble, does the unidirectionality (*i*.*e*., preference of one direction of rotation) of each individual molecule become evident. Among the ten rotating molecules in this surface area, only one molecule exhibits rotations in both directions while all the others maintain their direction of rotation, either clockwise or anticlockwise, over subsequent events.

To reveal the reason for this molecule-specific unidirectionality, we invoke the chirality of the molecules. This is known to invert the direction of rotation since in solution *R-* and *S-*enantiomers rotate in opposite directions within the molecular motor itself.^44^ The same image as in Figure 4b is plotted in Figure 4c with a multicolor contrast to highlight the chirality of each individual molecule (*R* or *S*, as indicated) with respect to the surface. The assignment is based on a recent study with experimental and simulated STM images that revealed characteristic appearances of each enantiomer: The approximate symmetry axis, which is shown as dashed red line for two molecules in Figure 4d and e, serves to distinguish between the two enantiomers.^31^ By comparing these enantiomers with Figure 4b, it becomes clear that the chiral state, *i*.*e*. enantiomer *R* or *S*, defines the direction of rotation: *R*-enantiomers of the MM1 molecule rotate clockwise (CW) and *S*-enantiomers rotate anticlockwise (ACW). The unidirectionality of these processes can be quantified as , and we determine ξ_MM1–*R*_ = −0.88 (*R*-enantiomer of the motor molecule MM1) and ξ_MM1–*S*_ = +0.96 (*S*-enantiomer) (Table 1). Among the 132 molecular rotations considered for this analysis, only 10 (*i*.*e*., 8%) happened directly below the STM tip. Statistically, no difference in the directionality is observed compared to remotely activated rotations. Hence, unidirectional rotation of the molecular motors is chirality-specific for each of the two enantiomers.

**Table 1:** Statistics of the Rotation Events for MM1 and MM2 Molecules, Each Separated for the Two Enantiomers, *R* and *S*^a^

Molecule	Enantiomer	*N*_total_	*N*_ACW_	*N*_CW_	*ξ*	CI (95%)
MM1	R	84	5	79	–0.88	(−0.96, −0.73)
	S	48	47	1	+0.96	(+0.78, +0.999)
MM2	R	83	10	73	–0.76	(−0.88, −0.58)
	S	55	47	8	+0.71	(+0.47, +0.87)

a *N*_total_: total number of molecules. *N*_ACW_: number of molecules rotating anticlockwise (ACW). *N*_ACW_: number of molecules rotating clockwise (CW). ξ: unidirectionality (see text). CI (95%) gives the 95% Clopper–Pearson confidence interval of the directionality.

By tracking the very same molecule over a sequence of rotations (Figure 4a), we also observed that the molecule stays in place during these rotations and no translation occurs (note that the same surface area is imaged as can be seen from the molecular trimer at the right). Close inspection of the rotations, including a fixed reference point on the surface nearby in the same image, reveals that there is a pivot point in the molecule that does not translate during the images (see Figure S6). Comparison with the simulated images suggests that this point is located at or close to the sulfur atom (see Figure S4). This interpretation is supported by calculations of the spatially resolved interaction between a single MM1 molecule and the Cu(111) surface underneath, which show that the S–Cu(111) interaction is the strongest within the molecule–surface system, thus representing a pivot point during rotation (see Figure S7).

Regarding the mechanism for molecular rotation, there are in principle two options:

(1) The motion is caused by activation of the motor unit in the molecules. This fits to the observed voltage threshold of about 0.5 V that agrees with the excitation voltages ∼0.5 V in a previous study with Feringa motors on Cu(111) that were assigned to resonant tunneling into the LUMO of the molecule and the formation of a negative ion resonance.^27^ Since the sulfur atom in the stator unit (see Figure 1a) acts as a pivot point as described above, it must be the rotor part of the molecule that rotates while the stator remains adsorbed on the surface. By assuming a propeller-like rotation of the rotor part, this should lead to a substantial change of the molecular appearance, because the methyl group (that points upward after molecular deposition; see Figure S4) would point toward the surface after a 180° rotation. This would result in a very different adsorption conformation and consequently appearance in STM images.^31^ However, this is not observed here as the molecule appears exactly the same after each rotation. Hence, it seems unlikely that motion of the molecular rotor unit is the underlying mechanism for the observed unidirectional rotations.
(2) The motion is not related to the motor mechanism, but results from a molecular excitation combined with an asymmetric potential energy surface. It is known that unidirectional rotation can occur in the absence of a motor unit and be independent from local deformations of the potential energy surface due to the STM tip^17^—an effect that is certainly irrelevant here as molecular activation is done remotely at distances up to about 100 nm (Figure 3b). Instead, it has been reported that unidirectional molecular rotation on a surface can be induced—in the absence of a motor unit—*via* molecular excitation^14,18^ in combination with an asymmetric potential energy landscape. An impressive example was reported recently by Stolz et al., who observed a clear unidirectionality of 97% with a simple acetylene molecule, thus in the absence of any motor unit, adsorbed on a chiral Pd_3_ cluster.^18^ We believe that a similar process is present in our case: The molecule is vibrationally excited out of equilibrium by inelastic scattering of the tunneling electrons, thus being lifted out of the potential well to a vibrational mode that couples to the rotational coordinate. After energy dissipation, the molecule drops down into the potential well, which—due to its asymmetry—results in unidirectional rotation. Accordingly, a mirrored potential energy landscape should cause the other direction of rotation. This fits precisely to our observations where the chirality of the molecule defines the preferred direction of rotation, because the potential energy pathway (along the rotational angle) is simply mirrored for the two enantiomers. Note that the remote excitation of the molecules excludes the possibility that a chiral STM tip^45^ could affect the rotation.

This assignment opens the question why the motor is not activated—in contrast to Ernst, Feringa and co-workers who studied a different chemical structure, but incorporated the same type of (Feringa) motors and used the same Cu(111) surface.^27^ We would like to point out that our observations do not exclude the possibility to excite the motor unit, but they are rather a result of different activation barriers. In other words, the motor might be excited, but the pure rotation of a molecule that maintains its conformation is energetically favored. Thus, a “rigid” rotation of the molecule occurs before a propeller-like rotation of the motor unit can take place. In fact, our calculations show that the energy barrier for a pure rotation of the MM1 molecules on the surface is about 150 meV (see Figure S8), while the adsorption energy of the rotor part is much higher, *i*.*e*., about 1.1 eV.^31^

This interpretation agrees with another result of our study, using a slightly altered molecular motor (MM2, Figure 1b) that is completely equal, but lacks the methyl group. It is well established that such a change affects the unidirectionality of the motor rotation where MM2 has no neighboring stereogenic center and therefore does not exhibit unidirectionality in solution.^46^ Thus, MM2 was designed as a “control molecule” that does not act as a unidirectional motor when in solution since the two directions of motion are not diastereomeric with respect to each other. This diastereomeric difference in left- vs right-handed rotation is the factor that induces the unidirectional rotation in MM1 when in solution. However, the situation changes upon adsorption of MM2 on the Cu(111) surface. In analogy to MM1, we have performed manipulation experiments with the MM2 molecule. The results (Table 1) demonstrate enantiomer-specific unidirectional rotation on the surface with the *R*-enantiomer rotating preferentially clockwise and the *S*-enantiomer preferentially anticlockwise. Hence, MM2 molecules rotate with a strong preference for one direction, depending on their chirality. We therefore assign the behavior to the same mechanism as for MM1: the surface-bound rotation is induced by molecular excitation in an asymmetric potential well and does not require a motor unit. Therefore, the difference between the solution activity for MM2 and its activity when surface-bound is striking. Nonetheless, the unidirectionality of surface-bound MM2 is less pronounced than in surface-bound MM1 (see Table 1): ξ_MM2–*R*_ = −0.76 (*R*-enantiomer) and ξ_MM2–*S*_ = +0.71 (*S*-enantiomer). We tentatively assign the reduced unidirectionality to a less distinct asymmetry of the potential energy surface.

## Conclusions

We have excited the rotation of molecular motors on a Cu(111) surface that remain in place *via* a chemical molecule–surface bond that acts as a pivot point. While the two directions of rotation seem to be equal when averaged over many molecules, it turns out that they are chirality-specific for each individual molecule. However, the fixed pivot point and the unchanged molecular conformation after a rotation suggest that the motor does not cause the motion. Instead, it seems more plausible that the molecule is vibrationally excited, which results in unidirectional rotation in an asymmetric potential energy landscape. This interpretation agrees with the results obtained from a control molecule that lacks unidirectionality in solution, but rotates unidirectionally on the Cu(111) surface. The reason for the missing motor activity seems to be the different activation barriers. Calculations show that the barrier for a pure “rigid” rotation is much smaller than that for a propeller-like motor activation. It therefore appears reasonable to conclude that nonmetallic surfaces are more suitable for the study of molecular motors, because aromatic systems on metals exhibit rather high adsorption energies and thus “stick” to the surface, rendering a motor rotation difficult and favoring other channels with lower barriers. Additionally, the potential energy landscape of a motor molecule should be less altered with respect to the gas phase on these surfaces as compared to metals.

## Methods

Experiments were performed under ultrahigh vacuum conditions in a low temperature STM (Createc). The Cu(111) substrate was cleaned by repeated Argon sputtering and annealing cycles, followed by deposition of molecules from a Knudsen cell (at a temperature of ∼375 K) onto the sample kept at room temperature (if not specified otherwise), with a typical coverage of about 0.1 monolayers. Subsequently, the sample is transferred into the cold STM (5 K) for imaging. Images were taken in the constant-current mode, applying the bias voltage to the sample while the tip is grounded. The molecular dynamics were then initiated by voltage pulses on a large number of molecules (analysis details are presented in the Supporting Information).

## Supporting Information Available

Analysis of molecular rotations using image recognition, small-angle rotations, calculations of MM1 and MM2 adsorbed on Cu(111), rotation dependence on the lateral molecule-tip distance, pivot point during molecular rotation, bonding strength (PDF)
