# Supplementary material for: Chirality-Specific Unidirectional Rotation of Molecular Motors on Cu(111)
Source: ACS Nano. 2023 Feb 9;17(4):3958–65. doi: 10.1021/acsnano.2c12720 (PMC9979643; doi:10.1021/acsnano.2c12720)
Supplement: Supplementary file 1 — nn2c12720_si_001.pdf [file nn2c12720_si_001.pdf]

Chirality-Specific Unidirectional Rotation of Molecular Motors on Cu(111)

Monika Schied,[1△] Deborah Prezzi,[2*] Dongdong Liu,[3] Stefan Kowarik,[1]

Peter A. Jacobson,[1△] Stefano Corni,[2,4] James M. Tour,[3*] and Leonhard Grill[1*]

1) Department of Physical Chemistry, Institute of Chemistry, University of Graz, Heinrichstraße 28, 8010 Graz, Austria

2) Nanoscience Institute of the National Research Council (CNR-NANO), via G. Campi 213/a, 41125 Modena, Italy

3) Departments of Chemistry and Materials Science and NanoEngineering, the Smalley Institute for Nanoscale Science and Technology, the Welch Institute for Advanced Materials and the NanoCarbon Laboratory, Rice University, Houston, Texas 77005, United States

4) Dipartimento di Scienze Chimiche, Università di Padova, Padova I-35131, Italy

*) Corresponding authors: deborah.prezzi@nano.cnr.it (D.P.), tour@rice.edu (J.M.T.) and leonhard.grill@uni-graz.at (L.G.)

△ Present addresses: Elettra-Sincrotrone Trieste S.C.p.A., Strada Statale 14 Km 163.5, Trieste, 34149 Italy (M.S.) and School of Mathematics and Physics, The University of Queensland, Brisbane, Queensland 4072, Australia (P.A.J.)

Contents

1. Analysis of molecular rotations using image recognition S2
2. Small-angle rotations S2
3. Calculations of MM1 and MM2 adsorbed on Cu(111) S5
4. Rotation dependence on the lateral molecule-tip distance S6
5. Pivot point during molecular rotation S7
6. Bonding strength S8
7. References S9

1. Analysis of molecular rotations using image recognition

The angle of rotation of MM1 is obtained by manually measuring the orientation of each molecule before and after the rotation with the software Gwyddion.[1] Taking the difference of the orientation angle of two consecutive images, the rotational angle is obtained. The rotational angle and therefore the sense of rotation is defined as the smaller angle between the two orientations. For example, a rotation of 120° could also be described as a rotation of -240° (in the opposite direction), but only values up to ±180° are considered.

In order to understand the directionality of the rotation of MM2, sequences of alternating images and pulses on the same molecule up to ca. 200 pulses have been recorded. The orientation of the molecule in each subsequent image is then determined using an image recognition neural network that takes STM images containing a single molecule as input and outputs the single variable of the molecular rotation angle. For the neural network architecture, we used a VGG16 convolutional neuronal network[2] implemented in python using the tensorflow framework.

From one sequence of images all images with the molecule in one and the same orientation are selected as basis for the training data. A cropped 256 px × 256 px section of the original 512 px × 512 px image with a single molecule is used, not only because neural network training is faster with smaller input data, but more importantly, there is usually some other adsorbate or defect in the image, which we exclude. The images are filtered with a fast Fourier transform (FFT) filter and the z values (apparent height) are scaled to the interval [0; 1] by subtracting the minimum and dividing by the (new) maximum value. Using a threshold of 0.1 the background (*i.e.* all values below 0.1) is set to zero. This ensures that the neuronal network is not trained to recognize noise in the image or the Cu(111) surface state instead of the actual molecular orientation.

Synthetic training data is generated by rotating the images in 0.5° steps and shifting the position of the molecule within the image up to ten pixels in random directions. The latter step is yet again to ensure training solely on the rotational angle, but not on the position of the molecule within an image (which is similar for one specific sequence, but may vary for other series). The training is carried out using gradient descent with back-propagation.[3] The actual input data (*i.e.* the full sequences) are pre-treated in the same way: cropping, FFT filtering, re-scaling to [0; 1]. The neural network then computes the orientation of the molecule in each image. In the same way as for MM1, the rotational angle is the difference between the orientation angles before and after the rotation.

2. Small-angle rotations

Besides the large rotations discussed in the main text, small rotations below 20° are observed. This is exemplarily shown for a series of three consecutive STM images in Fig. S1a. Between the images, voltage pulses of -350 mV have been applied to the spot marked with a yellow star (not shown in the second image). The red solid lines indicate the orientation of the molecule in each image. The dashed lines mark the molecular orientation in the respective previous image. Starting from the first image in the top panel, the molecule rotates clockwise by about 15°. In the second step, it rotates back to its original position. Notably, the general appearance of the molecule does not change. Upon more pulses, the same movement – back and forth – can be observed (but never two consecutive

rotations in the same direction). This back-and-forth movement is independent of the bias voltage and the pulse position (provided it moves at all).

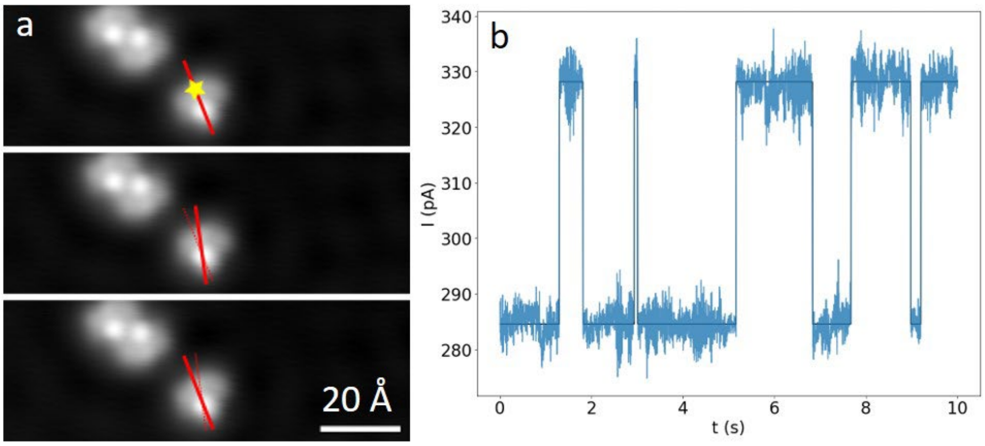

Figure S1: Consecutive images (-50 mV, 60 pA) of a motor molecule showing a small rotation of about 15° induced by voltage pulses of −350 mV. The yellow star marks the pulse position, the red solid and dashed lines indicate the current and previous orientation, respectively. (b) The current recorded during a voltage pulse. High and low current correspond to the two orientations seen in (a).

During the voltage pulse, the tip position is constant, including its height (feedback switched off). As the molecule is moving between two positions, a different part of the molecule will be below the tip and therefore a different tunnelling current is measured. The recorded current (Fig. S1b) exhibits two distinct levels, corresponding to the two orientations of the molecule seen in the image series in Fig. S1a. This so-called telegraph noise indicates that the underlying process is reversible.

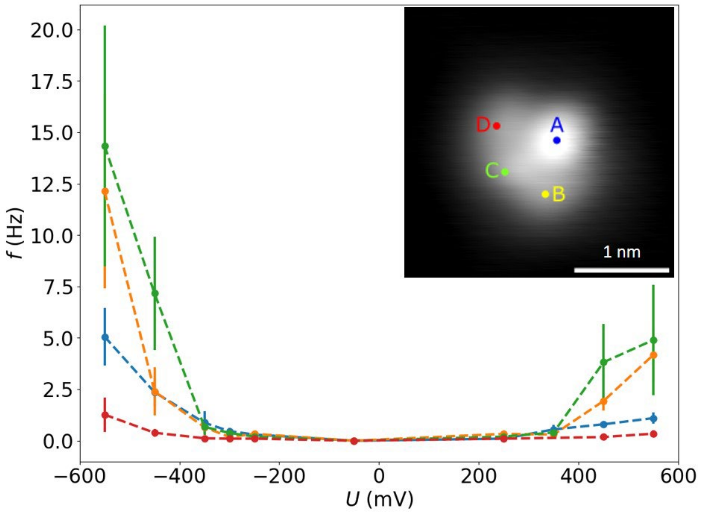

Figure S2: Switching rate depending on pulse voltage and position (as marked in the inset; 60 pA, −50 mV). The onset voltage is at about ±250 mV.

In order to do a systematic investigation, voltage pulses have been applied to a molecule on four distinct points a to d as shown in the inset of Fig. S2. On each position, multiple pulses with several seconds duration with a varying bias voltage between 50-550 mV have been applied.

With the algorithm from Yuzhelevski *et al.*,[4] the telegraph noise signal is analysed in the time domain. Each point in the current signal is ascribed to one of the two levels. For example, the fit for the signal in Fig. S1b is displayed as solid black line. From this, the lifetimes τ_i and the current levels I_i of each state i can be computed easily. The switching rate, computed as $\tau_i{}^{-1}$, is shown in Fig. S2 for the four positions A to D. For low bias, no switching has been observed. The minimum voltage to induce any switching events is about ±250 mV. This is comparable to the onset of ±200 mV for the helix inversion reported for a nanocar with four motors as "wheels".[5] Therefore, the small rotation of the motors could potentially be understood as the helix inversion process. However, as the appearance of the molecules does not change, it is more likely a transition between two similar adsorption sites on the Cu(111) surface.

Note that there are also some molecules that do not switch under the described conditions. This becomes apparent by scanning with a high bias. If a molecule moves under the tip, there is a sharp discontinuity in the scan image. An example can be seen in Fig. S3b.

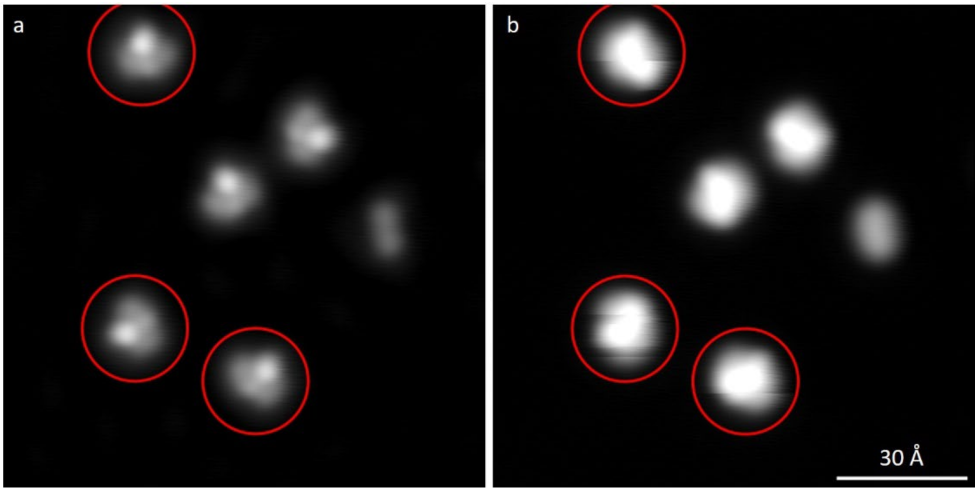

Figure S3: (a) Stable imaging of MM1 (-50 mV, 60 pA). No difference in appearance is observed between molecules with and without red circles. (b) When scanning the same surface area with a high bias of 1 V some molecules (marked with red circles in both panels) move under the tip, while other molecules are imaged stably. The movement seen as sharp lines corresponds to the same small rotation as observed during voltage pulses (cf. Fig. S1).

Three molecules marked with red circles change their orientation while scanning with a bias voltage of +1V. In contrast, the other two molecules never switch, even after multiple scans and with even higher bias voltage. Upon voltage pulses, the three circled molecules exhibit telegraph noise, whereas no change in current is observed for the other two. In the scan with a low bias of -50mV of the same area in Fig. 3a, all molecules have the same appearance. Also, the orientation on the surface does not differ from the bistable ones. The reason for this absent rotation is unclear. It might

be related to the adsorption and not of the molecule itself, because sometimes molecules rotate by larger angles during a voltage pulse. After this or a lateral manipulation, in which the molecule is moved across the surface using the STM tip, molecules that were originally stable can become bistable and vice versa.

3. Calculations of MM1 and MM2 adsorbed on Cu(111)

Ab-initio density functional theory (DFT) simulations were carried out to investigate the molecular adsorption on Cu(111) by using the Quantum ESPRESSO (QE) package,[6] which features a plane-wave, pseudopotential implementation of DFT. Dispersion forces were included by using an optimized vdW-DF-like[7] non-local exchange-correlation functional, where the GGA functional is replaced by an optimized Becke86b functional (optB86b-vdW),[8] which has been demonstrated to perform extremely well for the description of benzene adsorption on Cu(111).[9] Ultra-soft pseudopotentials were employed as available in the SSSP library.[10] The kinetic energy cutoff for the Kohn-Sham wave functions (charge density) was set to 35 (400) Ry. The molecule-surface system was modelled by using an orthorhombic supercell corresponding to a 8x4 repetition of a 4-layer slab of Cu(111) (optimized lattice parameter for bulk Cu: a = 3.59 Å). A vacuum region of at least 13 Å in the non-periodic direction (*i.e.* perpendicular to the slab) was introduced to prevent interaction between periodic images, while the in-plane irreducible Brillouin-zone was sampled with a 3x3 Monkhorst-Pack grid of k-points.

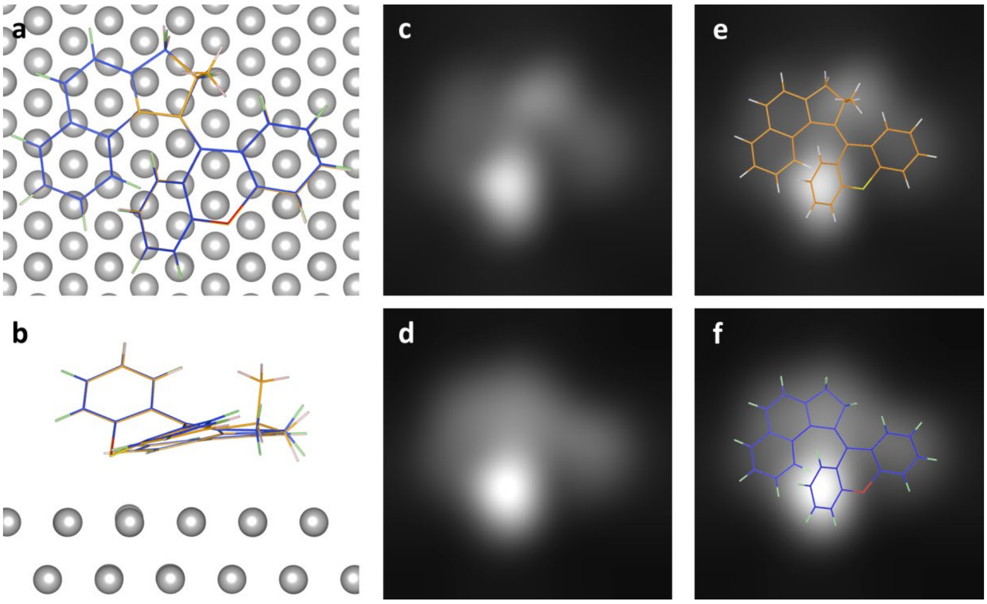

Figure S4: (a,b) Stick models of the calculated structures of MM1 (C in orange, H in white, S in yellow) and MM2 (C in blue, H in green, S in red) on Cu(111) (grey spheres). Top and side views are depicted in panels (a) and (b), respectively. (c-f) Simulated STM images of MM1 (c,e) and MM2 (d,f) molecules on Cu(111) (1.8 nm × 1.8 nm, map taken at -0.05 eV), which are shown for clarity both without (c,d) and with (e,f) superimposed atomic structure models. Experimental STM images of the two molecules are presented in Fig. 1c-d of the main text.

Ground state configurations were reached through a standard total-energy-and-forces optimization, as implemented in the pw.x code of QE. The atomic positions within the cell were fully relaxed until forces were less than 10 meV/Å. STM images of the optimized configurations were simulated within the Tersoff-Hamann approximation,[11] where the STM contrast is proportional to the local density of states (LDOS) evaluated at the position of a spherically symmetric (s-wave) tip. Image blurring was applied to the simulated STM plots to ease the comparison with experiments. Figure S4a displays the optimized structures of MM1 (C in orange, H in white, S in yellow) and MM2 (C in blue, H in green, S in red) on Cu(111), which have been plotted together to better highlight the differences. As can be seen, the molecular structure remains mostly the same in the presence/absence of the methyl group pointing upward, with minor adjustments around the 5-member ring.

4. Rotation dependence on the lateral molecule-tip distance

In order to understand the probability of inducing a rotation depending on the lateral distance from the tip position during a voltage pulse, a series of 14 voltage pulses (-850 mV, 120 s) is considered. Before and after each pulse, the very same surface area of 100×100 nm² is imaged (*cf.* Fig. 3a). The positions of all molecules in this area, as well as the coordinates of each voltage pulse, are recorded. Note that only monomers are considered: Due to their different chemical/physical environment dimers, trimers or other oligomers, as well as molecules adsorbed on step edges are excluded from this analysis.

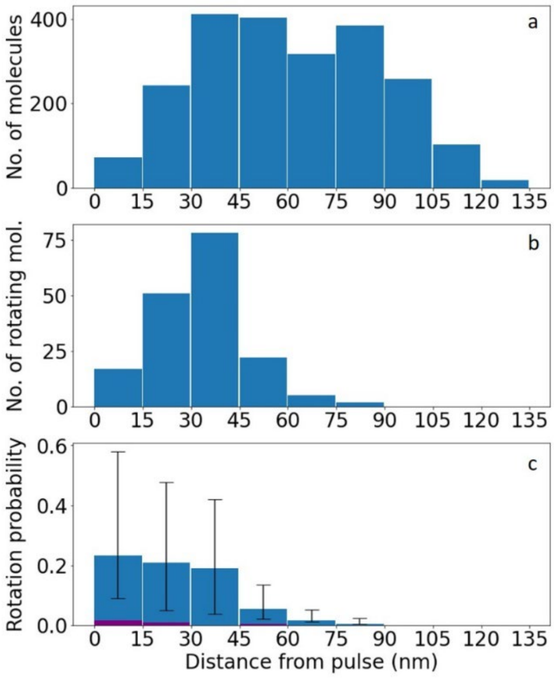

Figure S5: Evaluation of the distance dependent rotation rate. For 14 voltage pulses on the lower terrace (a) the total number of molecules and (b) of rotating molecules depending on the distance to the pulse position is measured. (c) The probability of rotation is the quotient of the data in (a) and (b). Error bars represent the 5 % confidence interval. The data in purple corresponds to pulses on the upper terrace.

For each pulse, the distance to all (single) molecules is computed, which results in the distribution of “available“ molecules depending on the distance from the tip as shown in Fig. S5a. This number can be understood as the number of molecules in a ring with a certain radius around the pulse position. Therefore, the number of molecules close to the tip is relatively low. The largest distances correspond to molecules in the diagonally opposite corner of the image with respect to the pulse position (*e.g.* a pulse in the top right corner as in Fig. 3a and a molecule in the bottom left corner). As only a small part of that “ring“ is covered by the square image, only few molecules are observed for very long distances.

In the next step, the rotating molecules are identified and their positions recorded, from which the distance from the tip during the voltage pulse is computed. In Fig. S5b the corresponding distribution is displayed. As this distribution not only depends on the likelihood of exciting a rotation, but also on the number of molecules available in a certain distance, the number of rotations observed in close proximity to the pulse position is relatively low. Therefore, the number of rotating molecules in a given distance is normalised by dividing it by the number of available molecules: $p_{rot} = N_{rot} / N_{mol}$. The result is the distribution shown in Fig. S5c (same as Fig. 3b). Assuming a binomial process - a molecule either rotates or stays stationary - the 95% Clopper-Pearson binomial proportion confidence intervals have been computed and are shown as error bars.

The same analysis has been performed for pulses on the upper terrace (top left corner in Fig. 3a) with molecules rotating on the lower terrace. The resulting values, displayed in purple in Fig. S5c, are clearly strongly reduced compared to excitation on the same terrace.

5. Pivot point during molecular rotation

Rotations show a pivot point, *i.e.* a fixed point that does not translate during rotation, as is evident in a sequence of rotations in the vicinity of a fixed reference point on the surface (Fig. S6). The images are aligned by the (stationary) trimer on the right and therefore show the exact same surface area. The red dot is on the same position relative to the top left corner in all five panels (as exemplarily indicated by the blue and green dashed lines in the first two panels). In each image, the red dot coincides with the same position on the molecule, despite the molecule being in different orientations. Therefore, this point acts as the pivot point. Comparison with the simulated images in Figure S4 suggests this point to be at or close to the sulfur atom of the stator.

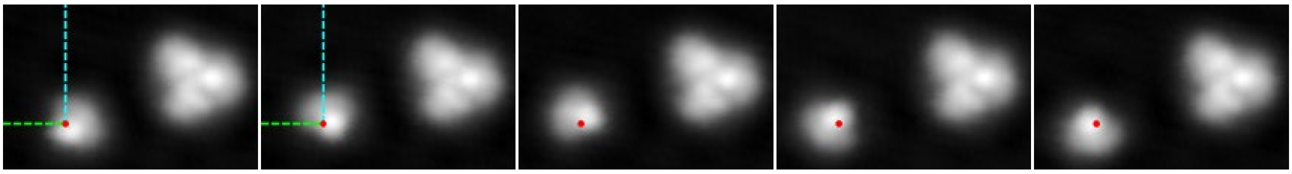

Figure S6: Sequence of STM images (60 pA, -100mV, 6.63 x 4.29 nm^2 each) of the same surface area for voltage pulse-induced rotations. All panels are aligned by the trimer on the right and the exact same position relative to the top left corner is marked with a red dot. Independent of the orientation of the molecule, this dot always coincides with the same position on the molecule. This point, which is nearby the sulfur atom of the stator, therefore acts as a pivot point of the rotation.

6. Bonding strength

In order to better understand the origin of this pivot point, we investigated by DFT calculations the spatially-dependent interaction of the molecule with the surface. In particular, we estimated the "local bonding strength" of the molecule by calculating the forces acting on each atom when the optimized molecule structure is rigidly shifted by a minimal value (*e.g.*, 0.1 Å) away from the surface along the *z* direction (*i.e.*, the direction perpendicular to the surface). The color map in panel a of Figure S7 displays the *z* component of the computed forces acting on each atom of the molecule (small colored dots) and of the topmost Cu layer (large colored dots), where the negative (positive) sign indicates a "restoring force" pointing toward (away from) the surface. The map clearly evidences that the S atom feels the strongest "attraction" toward the surface (small blue dot, *i.e.*, negative sign), paired to a similar effect on the surface Cu atom (large red dot, *i.e.*, positive sign), as also shown by the 3D stick models in panel b (two different lateral views; restoring forces are represented as blue arrows). The -C(H_2) atom of the pentagon in the rotor also displays a restoring force similar to that of the S atom, but this probably indicates an internal rearrangement of the molecule favored by the up-shift, since the forces on the Cu surface atoms underneath are negligible.

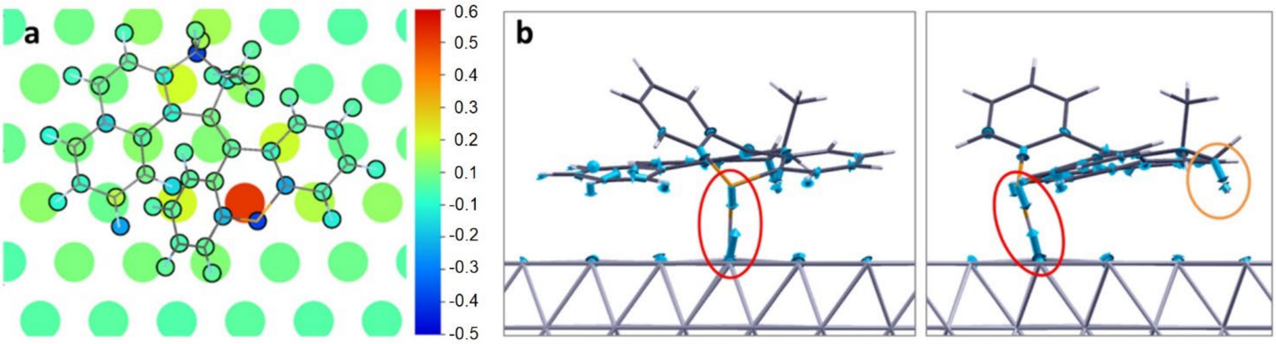

Figure S7: (a) Map of the z component (i.e., perpendicular to the surface) of the restoring forces acting on each atom of the molecule (small colored dots; the stick model of the molecule is superimposed for clarity) and on the topmost Cu layer (large colored dots) upon rigid shift of the molecule structure away from the surface (Δz = 0.1 Å). The color code of the small and large dots refers to the legend aside. (b) Two lateral views of the stick model of the molecule on Cu(111), where the restoring forces are represented as blue arrows in 3D.

This picture indeed supports the idea of the presence of a leading point-like attraction between the molecule and the substrate – corresponding to the S-Cu bond – that can act as pivot point during motion. Minimal rigid rotations (*e.g.*, θ = 1°) of the optimized molecule structure around an axis perpendicular to the surface – originating either in S or in the underlying Cu closest to S – indicate that the smaller restoring momentum is for rotations around the Cu atom. This preference can be understood in terms of the directionality of the S-Cu bond, and it would explain the 60° rotations observed experimentally, reflecting the symmetry of the rotation center. On this basis, we investigated the energetics of the system for rotation of the molecule about the Cu atom closest/bonded to the S atom. In view of the symmetry, we considered θ = 0° (*i.e.*, the optimized lowest energy structure, with S located in asymmetric bridge position) and θ = 30° (*i.e.*, the optimized

structure with S located in asymmetric hollow position), and explored the path connecting these two local minima to estimate the energy of the saddle point, which is found to be about 150 meV, as shown in Figure S8. The intermediate configurations are obtained by rigidly rotating the molecule at θ = 0° using steps of 5°; each of these intermediate starting configurations has been optimized by keeping the x coordinate of the S atom fixed, whereas the rest of the molecule and the topmost Cu layer are free to move: this is needed to explore the saddle point region and avoid the molecule to slip downhill toward one of the two local minima, θ = 0° and θ = 30°. We remark that the value found here only represents an upper bound of the energy barrier, since it has been obtained by exploring a predefined path.

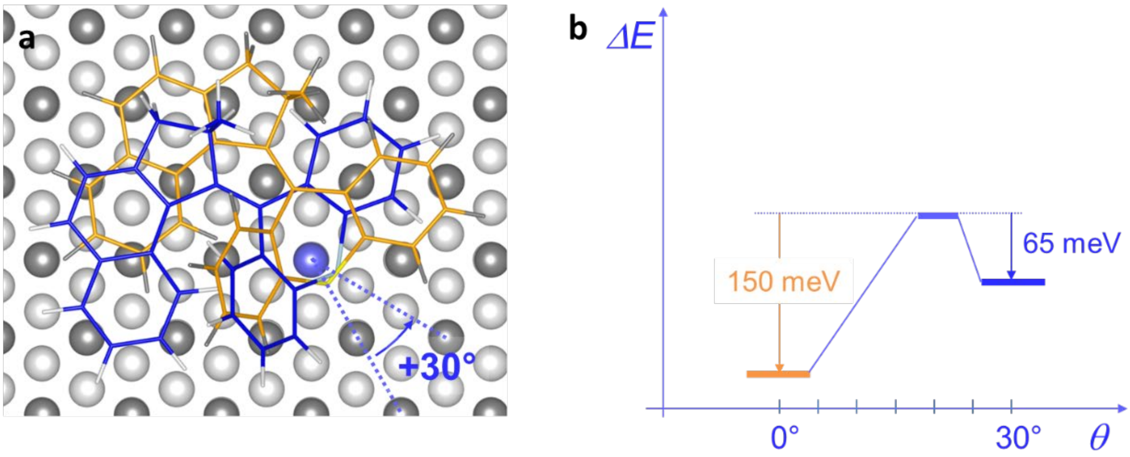

Figure S8: (a) Ball-and-stick model of MM1 molecules on the Cu(111) surface. The optimized lowest energy structure (θ = 0°; C atoms are in orange, S in yellow) is shown together with the local minimum at θ = 30° (C atoms are in blue, S in light blue). The substrate is represented by grey balls (dark grey for the topmost layer); the blue ball indicates the center of rotation. The path connecting these two minima is sampled to estimate the energy of the saddle point, as shown by the schematic in (b).

7. References

1. Necas, D.; Klapetek, P., Gwyddion: an open-source software for SPM data analysis. *Eur. J. Phys.* **2012,** *10* (1), 181-188.
2. Simonyan, K.; Zisserman, A., Very deep convolutional networks for large-scale image recognition. *The 3rd International Conference on Learning Representations (ICLR2015)* **2015,** *arXiv:1409.1556*.
3. LeCun, Y.; Boser, B.; Denker, J. S.; Henderson, D.; Howard, R. E.; Hubbard, W.; Jackel, L. D., Backpropagation applied to handwritten zip code recognition. *Neur. Comp.* **1989,** *1*, 541-551.
4. Yuzhelevski, Y.; Yuzhelevski, M.; Jung, G., Random telegraph noise analysis in time domain. *Rev. Sci. Instr.* **2000,** *71*, 1681-1688.
5. Kudernac, T.; Ruangsupapichat, N.; Parschau, M.; Macia, B.; Katsonis, N.; Harutyunyan, S. R.; Ernst, K.-H.; Feringa, B. L., Electrically driven directional motion of a four-wheeled molecule on a metal surface. *Nature* **2011,** *479*, 208-211.
6. Giannozzi, P.; Baroni, S.; Bonini, N.; Calandra, M.; Car, R.; Cavazzoni, C.; Ceresoli, D.; Chiarotti, G. L.; Cococcioni, M.; Dabo, I.; Corso, A. D.; Gironcoli, S. d.; Fabris, S.; Fratesi, G.; Gebauer, R.; Gerstmann, U.; Gougoussis, C.; Kokalj, A.; Lazzeri, M.; Martin-Samos, L.;

Marzari, N.; Mauri, F.; Mazzarello, R.; Paolini, S.; Pasquarello, A.; Paulatto, L.; Sbraccia, C.; Scandolo, S.; Sclauzero, G.; Seitsonen, A. P.; Smogunov, A.; Umari, P.; Wentzcovitch, R. M., QUANTUM ESPRESSO: a modular and open-source software project for quantum simulations of materials. *J. Phys.: Condens. Matter* **2009,** *21*, 395502.
7. Dion, M.; Rydberg, H.; Schröder, E.; Langreth, D. C.; Lundqvist, B. I., Van der Waals density functional for general geometries. *Phys. Rev. Lett.* **2004,** *92*, 246401.
8. Klimes, J.; Bowler, D. R.; Michaelides, A., Van der Waals density functionals applied to solids. *Phys. Rev. B* **2011,** *83*, 195131.
9. Carrasco, J.; Liu, W.; Michaelides, A.; Tkatchenko, A., Insight into the description of van der Waals forces for benzene adsorption on transition metal (111) surfaces. *J. Chem. Phys.* **2014,** *140*, 084704.
10. Prandini, G.; Marrazzo, A.; Castelli, I. E.; Mounet, N.; Marzari, N., Precision and efficiency in solid-state pseudopotential calculations. *Comp. Mater.* **2018,** *4*, 1-13.
11. Tersoff, J.; Hamann, D. R., Theory of the scanning tunneling microscope. *Phys. Rev. B* **1985,** *31*, 805.
